# Isolation of Mature (Peritoneum-Derived) Mast Cells and Immature (Bone Marrow-Derived) Mast Cell Precursors from Mice

**DOI:** 10.1371/journal.pone.0158104

**Published:** 2016-06-23

**Authors:** Steffen K. Meurer, Melanie Neß, Sabine Weiskirchen, Philipp Kim, Carmen G. Tag, Marlies Kauffmann, Michael Huber, Ralf Weiskirchen

**Affiliations:** 1 Institute of Molecular Pathobiochemistry, Experimental Gene Therapy and Clinical Chemistry (IFMPEGKC), RWTH Aachen University, University Hospital, Aachen, Germany; 2 Institute of Biochemistry and Molecular Immunology, RWTH Aachen University, University Hospital, Aachen, Germany; Universidade de Sao Paulo, BRAZIL

## Abstract

Mast cells (MCs) are a versatile cell type playing key roles in tissue morphogenesis and host defence against bacteria and parasites. Furthermore, they can enhance immunological danger signals and are implicated in inflammatory disorders like fibrosis. This granulated cell type originates from the myeloid lineage and has similarities to basophilic granulocytes, both containing large quantities of histamine and heparin. Immature murine mast cells mature in their destination tissue and adopt either the connective tissue (CTMC) or mucosal (MMC) type. Some effector functions are executed by activation/degranulation of MCs which lead to secretion of a typical set of MC proteases (MCPT) and of the preformed or newly synthesized mediators from its granules into the local microenvironment. Due to the potential accumulation of mutations in key signalling pathway components of corresponding MC cell-lines, primary cultured MCs are an attractive mean to study general features of MC biology and aspects of MC functions relevant to human disease. Here, we describe a simple protocol for the simultaneous isolation of mature CTMC-like murine MCs from the peritoneum (PMCs) and immature MC precursors from the bone marrow (BM). The latter are differentiated *in vitro* to yield BM-derived MCs (BMMC). These cells display the typical morphological and phenotypic features of MCs, express the typical MC surface markers, and can be propagated and kept in culture for several weeks. The provided protocol allows simple amplification of large quantities of homogenous, non-transformed MCs from the peritoneum and bone marrow-derived mast cells for cell- and tissue-based biomedical research.

## Introduction

Mast cells (MCs) are tissue-resident cells that are linked to the innate immune system. They are mostly known for their role in allergic and other inflammatory diseases [1,2]. Allergy is initiated by crosslinking of IgE-bound high-affinity receptors for IgE (FcεRI) by a specific antigen triggering MC degranulation [2]. In addition, MCs have a strategic location at the host-environment interface that predisposes them as a critical gate-keeper for starting early host defense against intruders [3]. On the other side, MCs are enriched in the tumour microenvironment of some carcinomas accelerating tumour progression, angiogenesis, epithelial-to-mesenchymal transition, and extracellular matrix degradation [4]. During the last years several MC-deficient mouse strains were established that were generated either by targeted mutations in the Kit or the stem cell factor gene or by introducing inducible or constitutive deficiencies under the use of different manipulating strategies [5].

Many studies have shown that MCs are crucial for the maintenance of tissue function, tissue homeostasis, and during all steps of tissue repair from the initial inflammatory reaction and proliferation of connective cellular elements to final remodelling of the extracellular matrix [6,7]. However, some effects of MCs are controversial and frequently opposite most likely due to the phenotypic heterogeneity of MCs in different tissues [8].

During the past decades, the unravelling of MC functions in many laboratories has been in the focus of MC research. Nevertheless, one of the major limitations is the difficulty to obtain large quantities of primary MCs for *in vitro* (e.g. for sensitization and signaling studies) and *in vivo* (e.g. for adoptive transfer experiments) purposes. Therefore, many studies have been conducted in immortalized MC cell lines (e.g., L138.8A, HMC-1) resulting in findings that must be interpreted cautiously due to activating mutations in key signalling components like Kit/KIT. In addition, there is a coincident opinion that the wide experimental possibilities that could be addressed by the accessibility of large quantities of purified and homogeneous MCs would allow addressing key questions of MC biology. Fundamental insights into differentiation of murine BMMC from bone marrow precursors and in isolation of resident peritoneal MCs were already performed decades ago [9,10]. The proposed protocols of these pioneering studies are used in many laboratories to isolate immature BMMCs or mature PMCs.

In principle, MCs can be derived from multipotent progenitor cells that are matured in specialized culture media, or directly isolated as functional MC from diverse tissues that are classified as tissue MC. Murine progenitor MCs can be derived from bone marrow (i.e. bone-marrow derived MCs, BMMCs) or foetal tissue (e.g. skin, liver, spleen) with high MC content. However, the generation of mature MCs is a long-lasting process that requires IL-3 and stem cell factor (SCF) or even more complex cocktails of cytokines and often results in questionable mixtures of cells with incomplete maturation [11]. Murine tissue MCs with a phenotype that is more consistent with connective tissue MCs can be isolated from the peritoneum and to a lesser amount from mucosa or skin [10]. Both MC entities may significantly differ from each other in functional terms and each by itself might have limited functional explanatory power in estimating general properties of MCs. As a consequence, the simultaneous availability and comparative analysis of culture-maturated and tissue-derived MCs from one biological source would be helpful to conduct experiments with more functional significance.

First protocols for the isolation of primary MCs required wasteful and time-intensive gradient centrifugation steps utilizing media containing sucrose, albumin, Ficoll, Percoll or Nycodenz [12–15]. Unfortunately, the cells that were isolated by these protocols showed morphological and functional changes such as loss of histamine and alteration of responsiveness against histamine-releasing agents and antigens. Also the isolation of MCs from tissue by enzymatic digestion protocols using collagenase and elastase resulted in cells with variable viability and purity [16]. Other protocols allowed the isolation of rat peritoneal MCs (PMCs) from a single peritoneal lavage resulting in high yield of MCs [17]. In addition, time consuming multistep separation techniques that combined enzymatic treatment, elutriation, magnetic CD14^+^ cell depletion (monocytes, macrophages) and cell sorting techniques with monoclonal antibodies directed against CD117/KIT were suitable to isolate and enrich MCs from human lung, skin, and uterus [18].

Here, we describe a two-step simple protocol suitable to simultaneously isolate murine mature MCs from the peritoneum and immature MC progenitors from the bone marrow. The resulting cells are highly homogenous and show the typical MC phenotype and expression of marker genes and can be propagated in culture.

## Experimental Procedures

### Instrumentation and equipment

A complete material list of all surgical instruments, laboratory equipment and sterile preparation utensils (scissors, forceps), plastic ware (Falcon tubes, 10 mL syringes, plastic Pasteur pipettes) that we use in our laboratory is given in S1 Table. Further equipment necessary are pre-cooled centrifuges and a standard cell culture including a sterile hood and a humidified incubator.

### Solutions

There are several solutions that must be prepared prior beginning of the protocol. All these solution that are listed in the following should be prepared in sterile fashion.

#### a) Gey’s solution A

35 g NH_4_Cl, 1.85 g KCl, 1.5 g Na_2_HPO_4_, 0.75 g K_2_PO_4_ x 2 H_2_O, 5.0 g D-glucose, 50 mg phenol red (all from Merck, Darmstadt, Germany) are added to 1 L of distilled H_2_O. Sterile filter and store in 500 mL aliquots at 4°C.

#### b) Gey's solution B

0.42 g MgCl_2_ x 6 H_2_O, 0.14 g MgSO_4_ x 7 H_2_O, 0.45 g CaCl_2_ x 2 H_2_O (all from Merck, Darmstadt, Germany) are added to 100 mL of distilled H_2_O. Autoclave and store in 50 mL aliquots at 4°C.

#### c) Gey's solution C

2.25 g NaHCO_3_ (from Merck, Darmstadt, Germany) is added to 100 mL of distilled H_2_O. Autoclave and store in 50 mL aliquots at 4°C.

#### d) Gey's working solution

To obtain the Gey’s working solution 70 mL of distilled H_2_O, 20 mL of Gey's solution A, 5 mL of Gey's solution B, and 5 mL of Gey's solution C are mixed and sterile filtered.

#### e) Phosphate-buffered saline (PBS)

8 g NaCl, 0.2 g KCl, 1.42 g Na_2_HPO_4_ and 0.27 g KH_2_PO_4_ (all chemicals were obtained from Merck) are added to 1 L of distilled H_2_O. Sterile filter and store in 500 mL aliquots at 4°C.

#### f) Ethanol, 70% (v/v)

700 mL absolute ethanol is mixed with 300 mL distilled H_2_O.

#### g) BMMC medium

RPMI 1640 medium, including 2 mM L-glutamine, was supplemented with 15% FCS, 1 x Penicillin/streptomycin (all from Gibco, ThermoFisher Scientific, Darmstadt, Germany), 0.07% β-mercaptoethanol, 10 mM HEPES and 1% of X63-Ag8-653-conditioned medium as a source of IL-3 (final concentration: ~ 30 ng/mL) that is necessary for proper MC differentiation and growth [9]. The myeloma cell line X63-Ag8-653 stably carries plasmids constitutively driving expression of large quantities of mouse IL-3 [19].

#### h) PMC medium

BMMC medium supplemented with supernatant derived from CHO cells that were transfected with an expression vector constitutively expressing murine SCF. The final concentration of SCF in the PMC medium as assessed by ELISA testing was adjusted to approximately 20 ng/mL.

#### Animals

All mice (C57BL/6J) were maintained under specific-pathogen-free conditions in the RWTH Aachen University Hospital Laboratory Animal Facility according to the guidelines of the Federation for Laboratory Animal Science Associations (FELASA). Animals were housed at 4–5 mice per cage in an animal room maintained at a constant temperature of 20°C, a relative humidity of 50%, and with a 12-hour on/off light cycle. The animals had free access to a standard mouse chow and trap water. For the performed experiments, female or male mice at age of 4–8 weeks that were bred in the RWTH Aachen University Hospital were taken. The protocol was approved by the author’s Institutional Animal Care and Use Committee (LANUV, Recklinghausen, Germany) and all experiments were conducted in accordance with the German federal law regarding the protection of animals and 'Guide for the Care and Use of Laboratory Animals' (National Institutes of Health publication 8^th^ Edition, 2011 [20].

#### Surgical procedures

**Fig 1:**
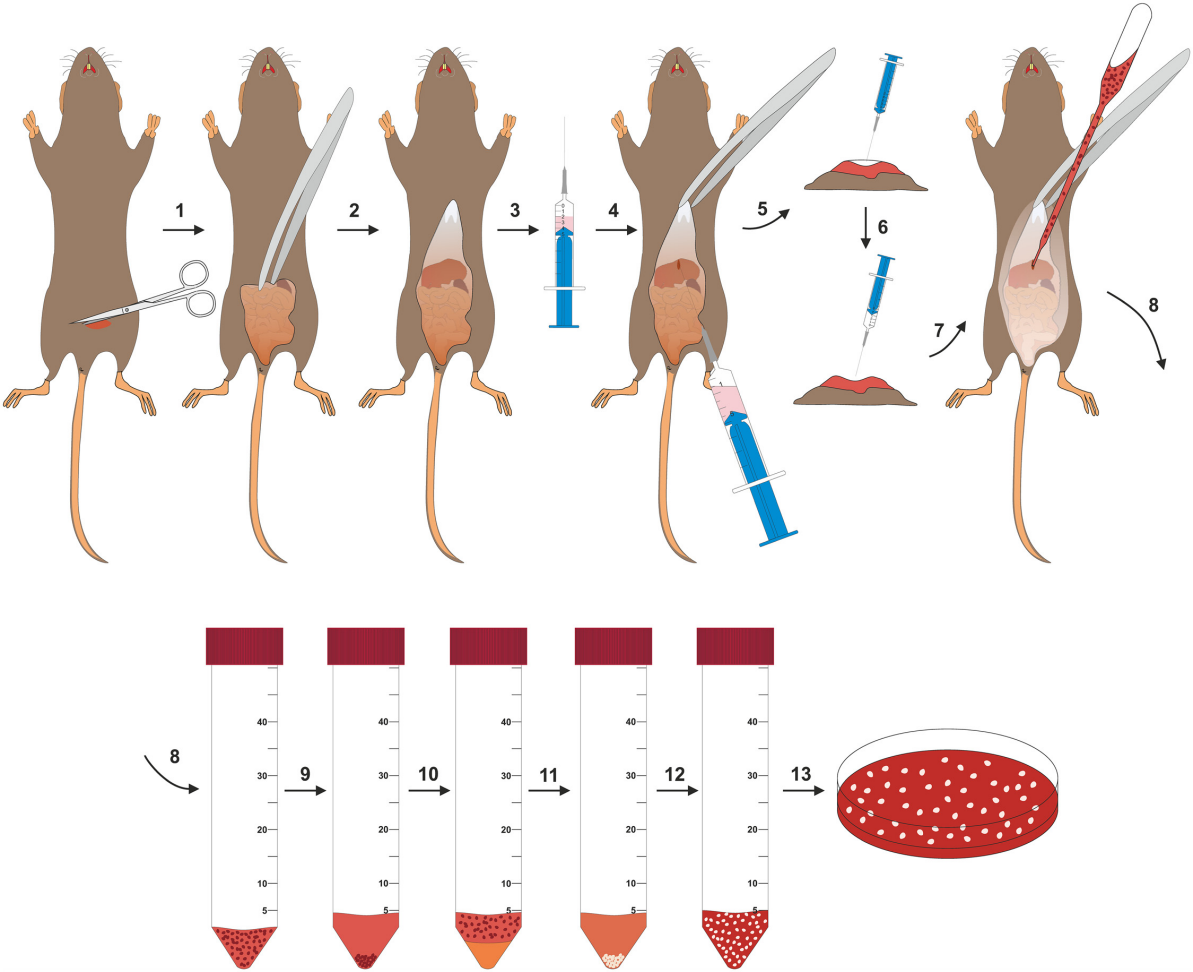
Schematic overview of the protocol for isolation of PMCs. Before starting with the isolation protocol, the mouse is anesthetized by inhalation of Isofluran and then sacrificed by cervical dislocation. In the first step of the isolation protocol, the abdominal skin is lifted up with forceps and an incision in the abdominal skin is made using scissors (1). The skin is fixed by forceps and torn towards the sternum, so that the cutis plus facia is separated from the lower fabric up to the top of the sternum to expose the abdominal wall (2). Using a 10-mL syringe that is filled with 3 mL of PBS and 2 mL of air (3), PBS and air is injected into the peritoneal cavity at the *linea alba* in direct proximity to the sternum (4–5). Thereafter, the mouse is carefully shaken in the palm of the operator (not shown). The mouse is then placed back on the operation table. Subsequently, the air that was brought in before is removed with a syringe (6). Subsequently, the cell-containing fluid of the peritoneal cavity is gently collected in a plastic Pasteur pipette (7) and placed into a Falcon tube (8). Cells are then pelleted by centrifugation (9). If the cell suspension shows a red colour due to contaminating blood cells, the resulting cell pellet is resuspended in Gey’s solution and left on ice for 5 min. For further purification, the cell suspension is underlain with fetal calf serum (10) and centrifuged (11). The final cell pellet is suspended in PMC medium (12) and plated into cell culture dishes (13).

Schematically, the outlined protocol can be divided into two steps. In the first step, peritoneal cells are flushed out from the animal’s peritoneal cavity (Fig 1). This cell population contains a small fraction (~ 1–3%) of PMCs that are enriched and amplified during prolonged cell culture periods, while other cell populations do not survive the selected cell culture condition. In addition, PMCs may emerge from MC precursors.

**Fig 2:**
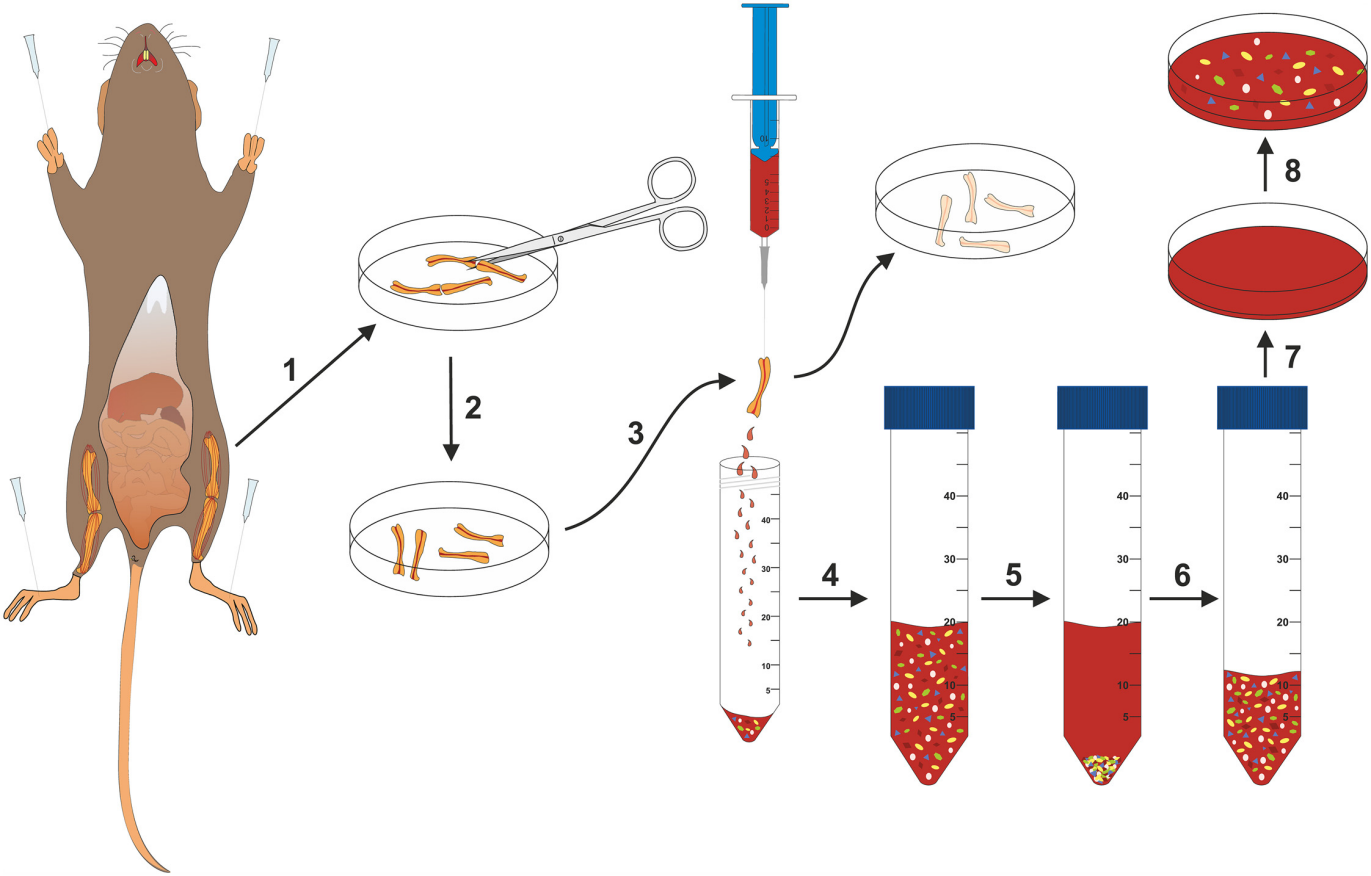
Schematic overview of the protocol for isolation of bone marrow cells. The hind legs are fixed, the skin of the legs is removed to the ankle, and the long bones of the legs of the fixed mouse are exposed by removing the muscle tissue (1). The legs are detached from the body at the hip joint and the ankle. The tibia and femur of each leg are separated at the knee joint (2), the bone ends are chopped off, and the bone marrow rinsed out with a syringe filled with BMMC medium (3) and collected in a Falcon tube (4). The cells are enriched by centrifugation (5) and the final cell pellet dissolved in BMMC medium (6) and added into Petri dishes filled with BMMC medium (7–8).

In the second step, the long bones of the legs are exposed and stem cells and progenitor cells are purged out from the tibia and femur with repeated injections of media with a syringe (Fig 2).

**Fig 3:**
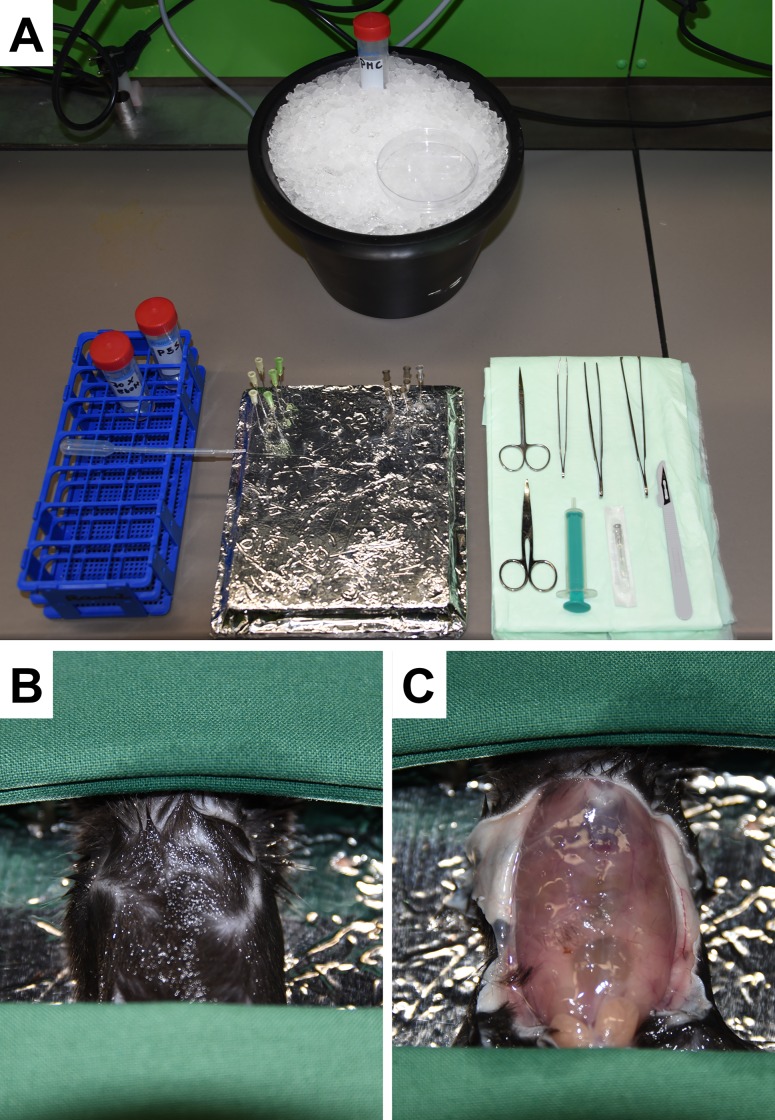
Experimental set up for isolation of mast cells. **(A)** A simple operational work area is prepared by wrapping an aluminium foil around a Styrofoam plate. All instrumentation that is necessary during the surgery is placed in clear order. **(B)** The sacrificed mouse is layered on the work area and the abdominal skin sterilized with 70% ethanol. **(C)** The abdominal skin is removed and the operation area (abdominal wall) exposed for further interventions.

In the following the different steps are successively described:

1. All surgical procedures were carried out under clean but non-sterile conditions. The surgical forceps and scissors were sterilized prior usage according to the guidelines necessary to perform surgeries in animals.
2. A Styrofoam plate is wrapped in aluminium foil and a number of needles needed for fixation of the mouse are prepared for use. This construction will serve as the workspace.
3. An appropriate inhalation anaesthetic such as isoflurane is placed ready to hand.
4. The necessary instruments (forceps, scissors, and plastic Pasteur pipette) and an ice bath are arranged in a manner that they are easily accessible. As a reservoir for the peritoneal lavage a 50 mL Falcon tube is placed on ice as well as a tissue culture plate to keep the long bones cold until proceeding with the purification in cell culture. (Fig 3A). A 1.5 ml tube is placed on ice for a tail biopsy if handling genetically modified animals to re-check the genotype.
5. The mouse is sacrificed by inhalation of an anaesthetics followed by cervical dislocation.
6. The mouse is placed on the workspace (Fig 3B) and the abdominal skin sterilized with a gauze swab that is moistened with a standard antiseptic. We prefer to use a solution of 70% ethanol.
7. An incision into the abdominal skin is made and the cutis plus facia is cut open crosswise at a length of approximately 3 cm with an 11.5 cm surgical scissor.
8. The cutis is separated from the lower fabric up to the top of the sternum using forceps (Fig 3C). In our experience, forceps that closely grabs the skin are most suitable for separation of the cutis.

### Isolation of peritoneal cells

**Fig 4:**
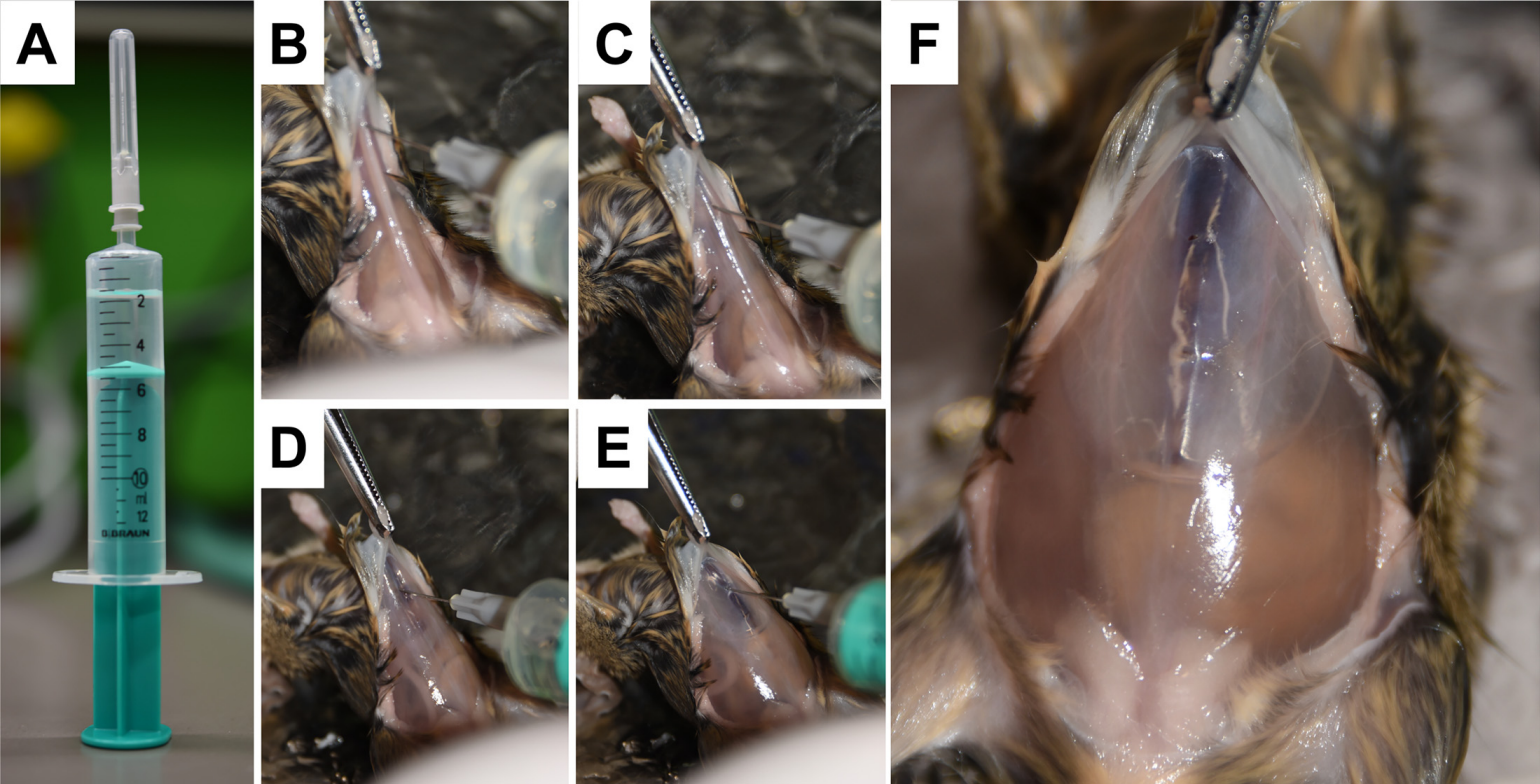
Ballooning of peritoneal cavity. **(A)** A syringe is filled with PBS (3 mL) and air (2 mL) and the content **(B-E)** injected into the peritoneal cavity of the mouse. **(F)** This procedure results in a ballooned peritoneal cavity.

**Fig 5:**
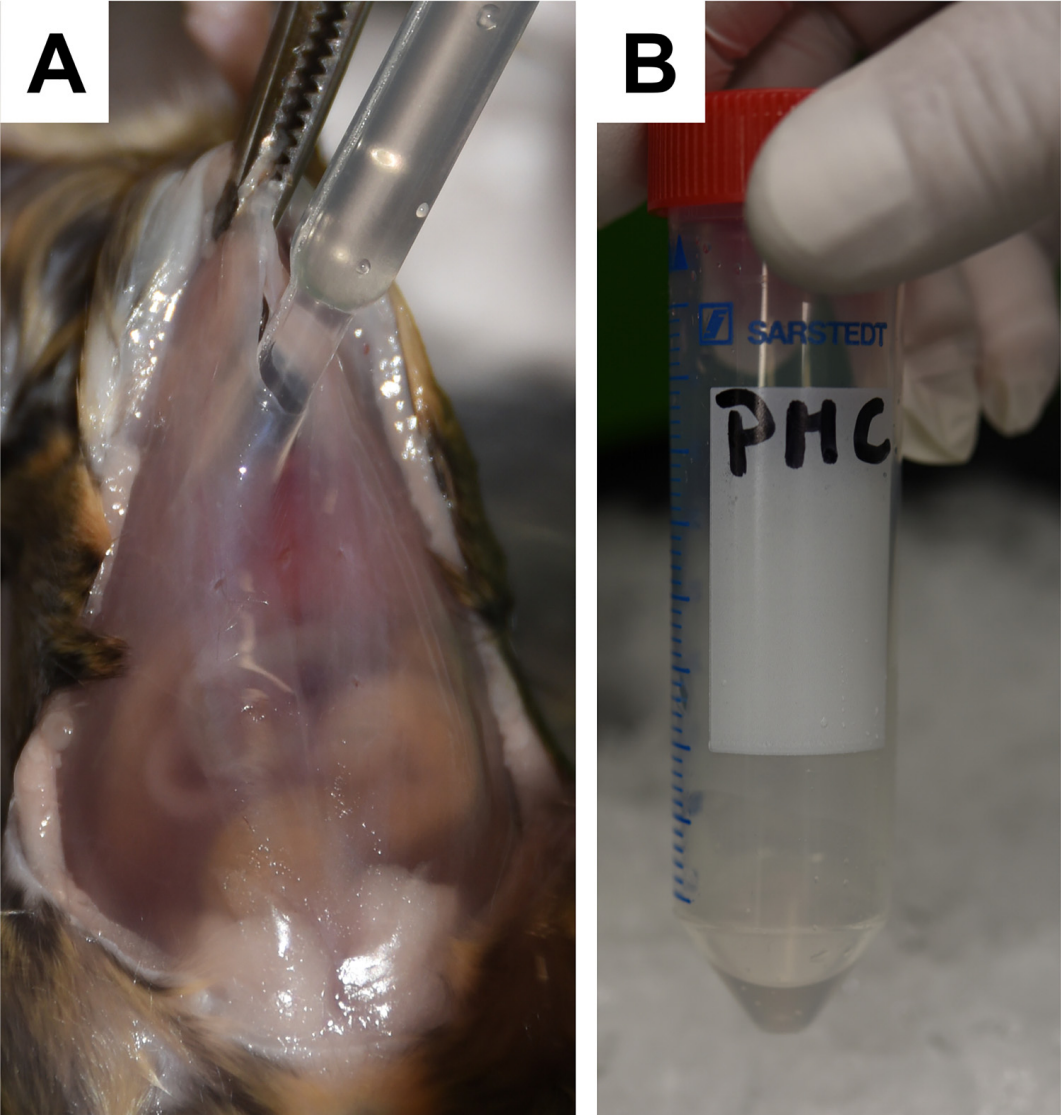
Extraction of peritoneal cell-derived mast cells. The mouse is carefully shaken in the palm of the operator, the air removed with a syringe and a small incision is made in the *linea alba* (not shown). **(A)** The PMC-containing solution is withdrawn from the peritoneal cavity using a Pasteur pipette and **(B)** placed into a sterile Falcon tube.

1. A 10-mL syringe is filled with 3 mL of PBS and 2 mL of air (Fig 4A).
2. The sternum is raised with a forceps and the content of this syringe is then slowly injected into the peritoneal cavity at the *linea alba* to prevent disintegration of vessels and leaking of blood into the peritoneal cavity (Fig 4B–4E).
3. This procedure results in a bloated cavity in which the subcutaneous tissue is separated from the internal organs and the peritoneal cavity is filled with PBS (Fig 4F).
4. The mouse is placed into a hand and the mouse body is carefully shaken several times. This proceeding will allow the cells to detach from tissue and transit into the PBS solution.
5. Thereafter, the sternum is lifted with a forceps and the air is removed by a syringe. To collect the cell containing PBS solution (~ 3 mL) an incision is made in the *linea alba* directly below the sternum. Thereafter, the cell suspension is pulled out from the peritoneal cavity with a plastic Pasteur pipette (Fig 5A). The cell containing solution (~ 3 mL) is filled into a Falcon tube and stored on ice until later use (Fig 5B). Note: The used plastic Pasteur pipette is perforated several times at the tip with a hot needle. This prevents attachment of highly vascularised organ surfaces to the pipette tip and thereby disintegration of vessels.

### Isolation of bone marrow-derived cells

**Fig 6:**
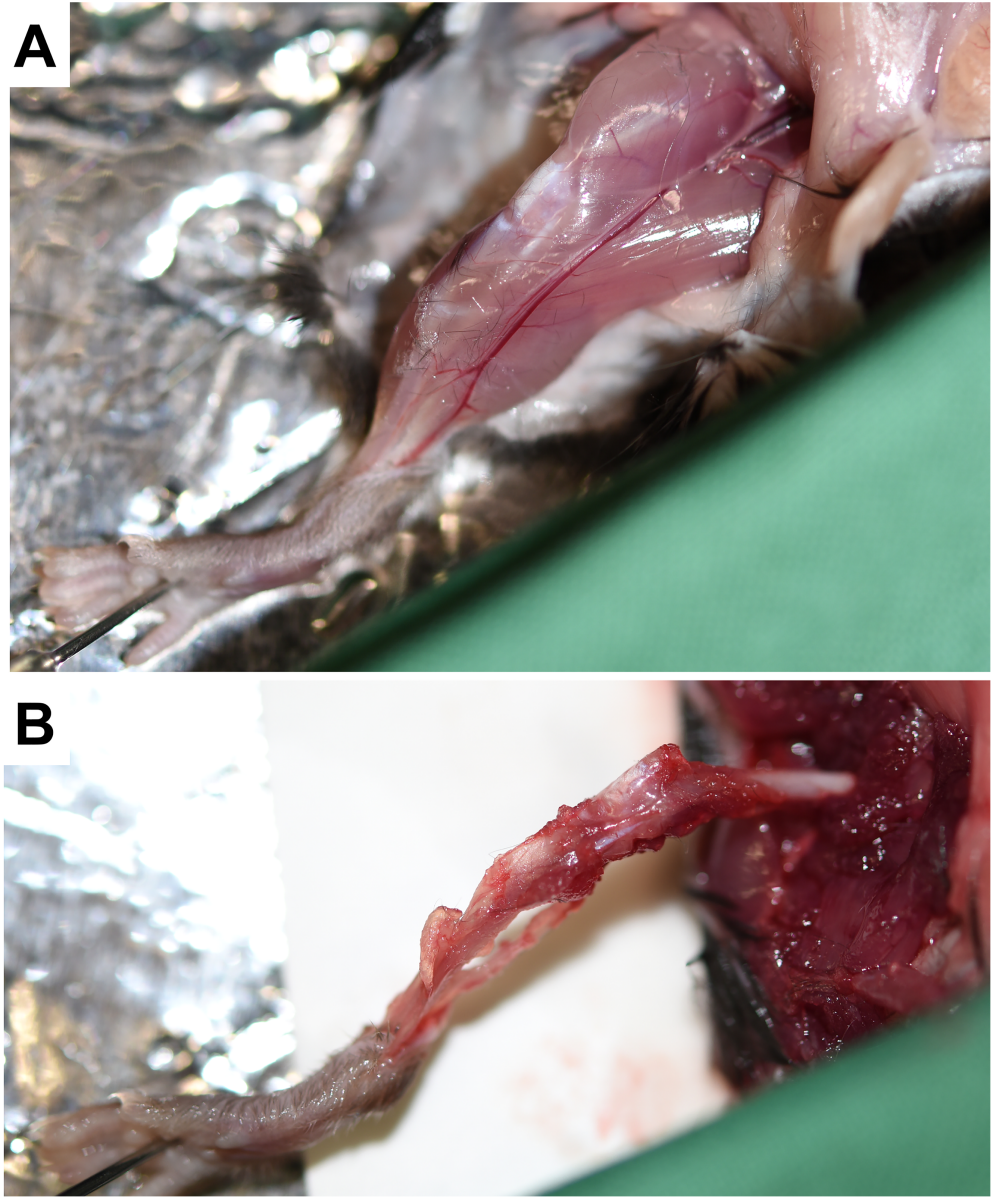
Exposure of the bones. The mouse extremities are fixed with needles for subsequent surgery steps (not shown). **(A)** Thereafter, the skin is removed to expose the legs and **(B)** the muscle tissue is separated and discarded.

**Fig 7:**
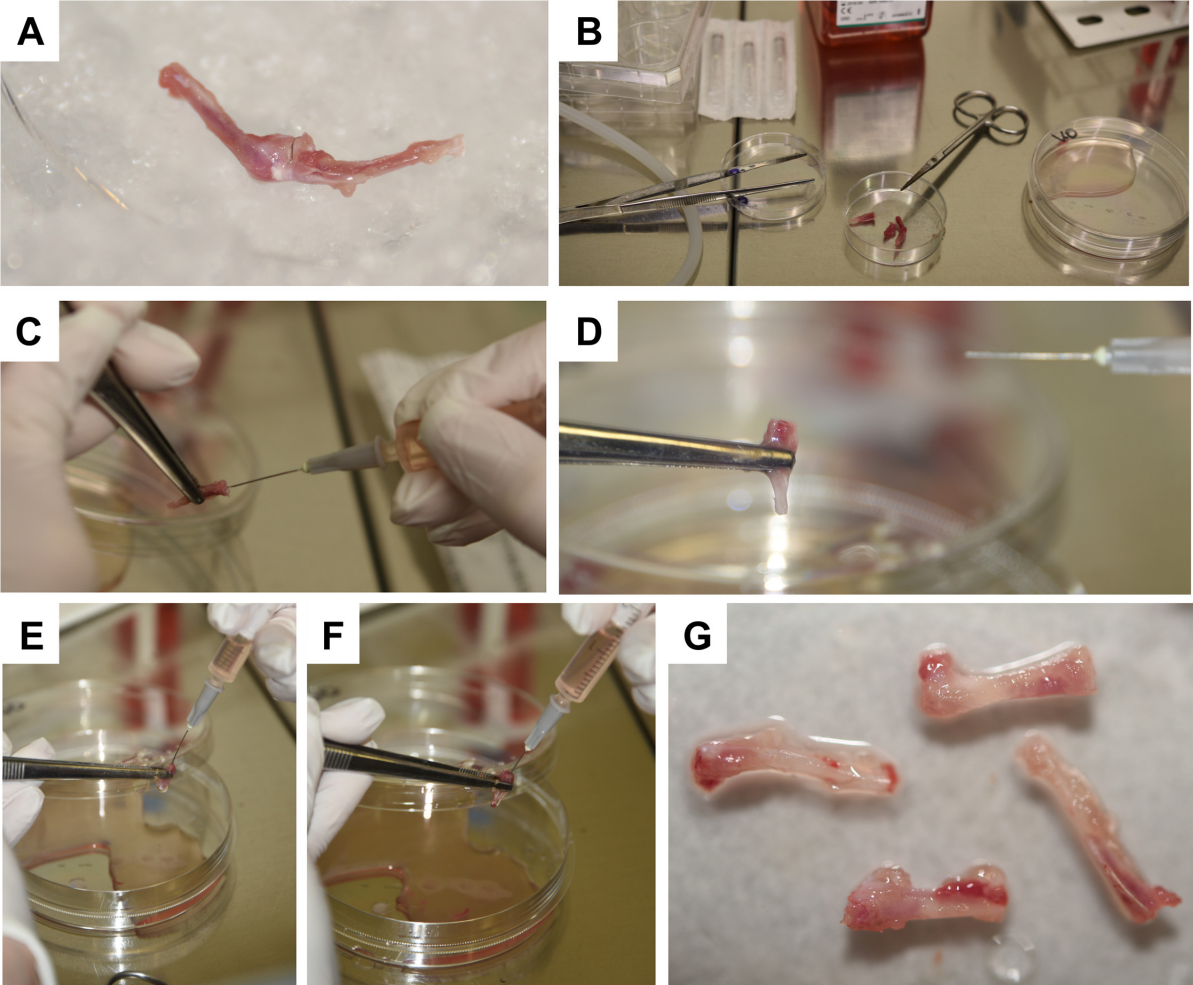
Harvesting of bone marrow cells and culturing of bone marrow-derived mast cells. **(A)** After disconnection of the legs from the body, the bones are finally put on ice and **(B)** the last steps of BMMCs preparation performed under a sterile cell culture hood. **(C-F)** The bone marrow from tibia and femur are flushed out of the bone by extensive rinsing with BMMC medium and collected. **(G)** The resulting hollowed and gnawed bones that have a pale appearance after removal of the bone marrow cells are discarded.

1. The mouse is then fixed on the workspace with four needles that pierce the four extremities.
2. The long bones of one leg of the fixed mouse are exposed by first removal of the skin from the hip to ankle and thereafter the muscles of the extremities are carefully removed with scissors without breaking the bones (Fig 6).
3. The long bones are removed from the body by releasing them from hip and ankle and placed into an iced cooled Petri dish (Fig 7A).
4. Thereafter, the long bones of the second leg are prepared in the same manner and placed into an iced Petri dish.
5. To prevent contamination of the bone marrow, all further steps in the cell isolation protocol are now performed under a sterile cell culture hood in which all necessary equipment (medium, cell culture plates, racks, syringe, needle, forceps, scissors, 50 mL Falcons) are ordered in clear arrangement (Fig 7B).
6. In the next step, the cells of the bone marrow are rinsed out. Therefore, the separated thighs and calves of each leg are successively fixed with a forceps and the end of each bone is removed with scissors to open the bone marrow cavity, followed by flushing each bone with 5 mL culture medium. Therefore, culture medium is flushed into both sides of each bone and the fluid collected either directly in a 50 mL Falcon tube (not shown) or in a Petri dish (Fig 7C–7F). In this step it is essential that the bone marrow is rigorously rinsed. At the end of this procedure, the hollowed and gnawed bones (Fig 7G) can be disposed.
7. The cell suspension (20 mL) is transferred into a Falcon tube that is placed on ice.

### Cell harvesting, enrichment, and differentiation

#### a) Peritoneal mast cells

**Fig 8:**
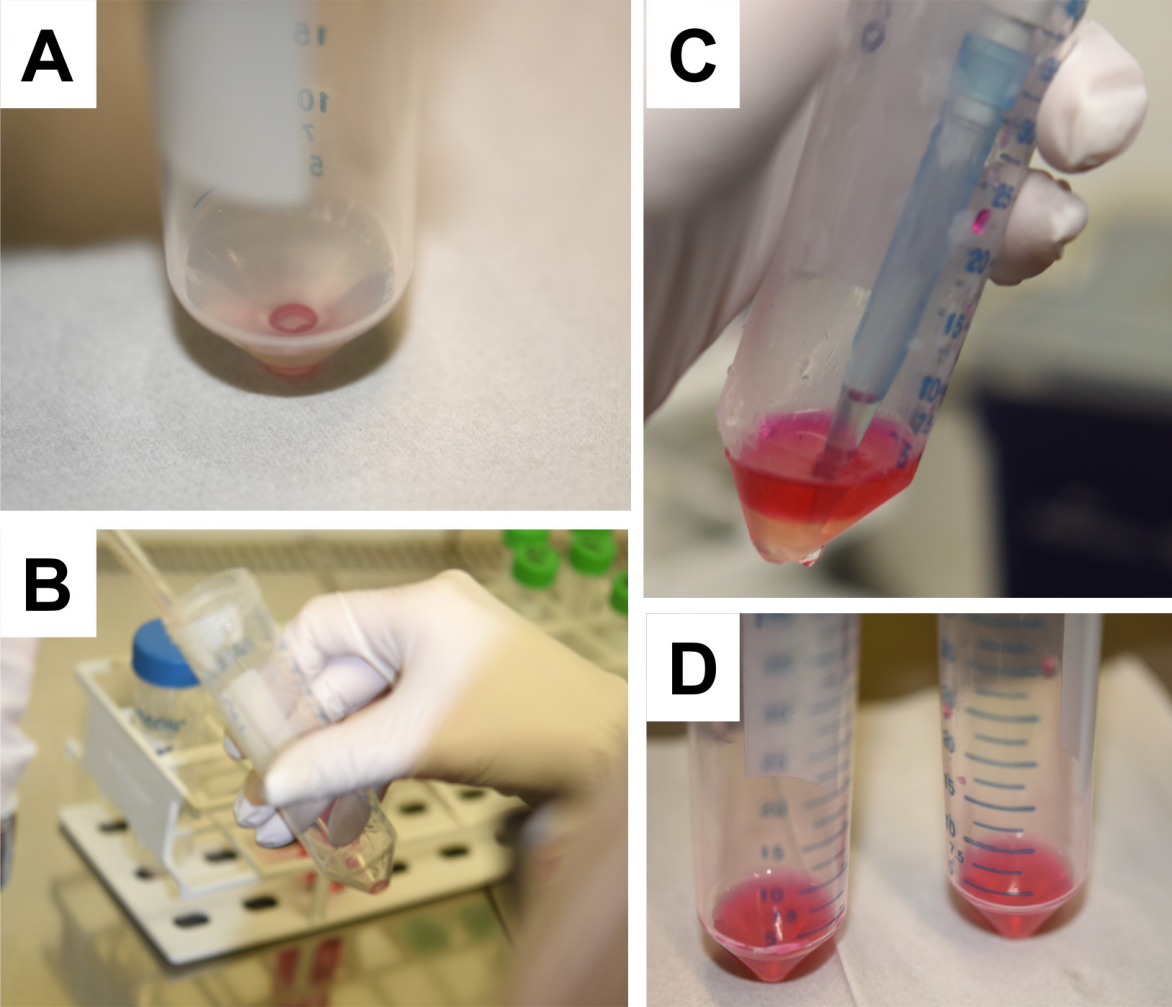
Preparation of PMCs for cell culture. **(A-D)** The isolated PMCs are enriched by centrifugation **(A)**, dissolved in Gey’s solution, left for 5 min on ice **(B)**, and underlain with foetal calf serum **(C)**. After centrifugation, the final cell pellet is suspended in PMC medium **(D)** for final cell cultivation.

1. If the retrieved cell suspension from the peritoneal lavage shows a red colour due to contaminating blood, cells were washed with Gey’s solution (proceed at step 2). If the PBS is clear directly proceed at step 5.
2. The ~ 3 mL cell suspension from step 5 in paragraph “Isolation of peritoneal cells” is centrifuged for 5 min at 1,200 rpm at 4°C.
3. After centrifugation, the supernatant is removed (Fig 8A) and the cell pellet resuspended in 2.5 mL Gey’s working solution (Fig 8B) and stored on ice for 5 min.
4. Then 2 mL of heat-inactivated foetal calf serum is carefully layered under the cell solution (Fig 8C).
5. The Falcon tube is then centrifuged for 5 min at 1,200 rpm at 4°C.
6. The supernatant is gently removed and the cell pellet dissolved in 3 mL PMC medium (Fig 8D) and platted into the well of a six-well plate.
7. The cell culture plate is then placed into a standard incubator at 37°C with a humidified atmosphere containing 5% CO_2_.
8. Fresh medium is added to the cells every third day of culturing. If cells reach an appropriate density, the cells are passaged. Cell enrichment for passaging can be done by removal of consumed medium by low speed centrifugation (1,200 rpm, 5 min at room temperature).
9. The enrichment and amplification of PMC should be completed after 2–3 weeks. Please note that the progress of differentiation and purity of the cell fraction can be determined by FACS analysis (see below).

#### b) Bone marrow-derived mast cells

1. The 20 mL cell suspension from step 7 in paragraph “Isolation of bone marrow-derived cells” is centrifuged for 5 min at 1,200 rpm at 4°C.
2. In the meantime, two Petri dishes were filled with sterile 6 mL BMMC medium.
3. After centrifugation, the supernatant is removed and the cell pellet dissolved in 12 mL BMMC medium.
4. 6 mL each of this cell solution is then transferred into the prepared Petri dishes.
5. Cell culture plates are then incubated in a standard incubator at 37°C in a humidified atmosphere containing 5% (v/v) CO_2_.
6. Fresh medium is added to the cells every third day of culturing. Cell enrichment for passaging can be done by removal of consumed medium by low speed centrifugation (1,200 x g, 5 min at room temperature).
7. The complete differentiation of BMMC should be completed after 4–6 weeks. Please note that the progress of differentiation can be monitored by FACS analysis (see below).

### Analysis of PMCs and BMMCs by light microscopy

**Fig 9:**
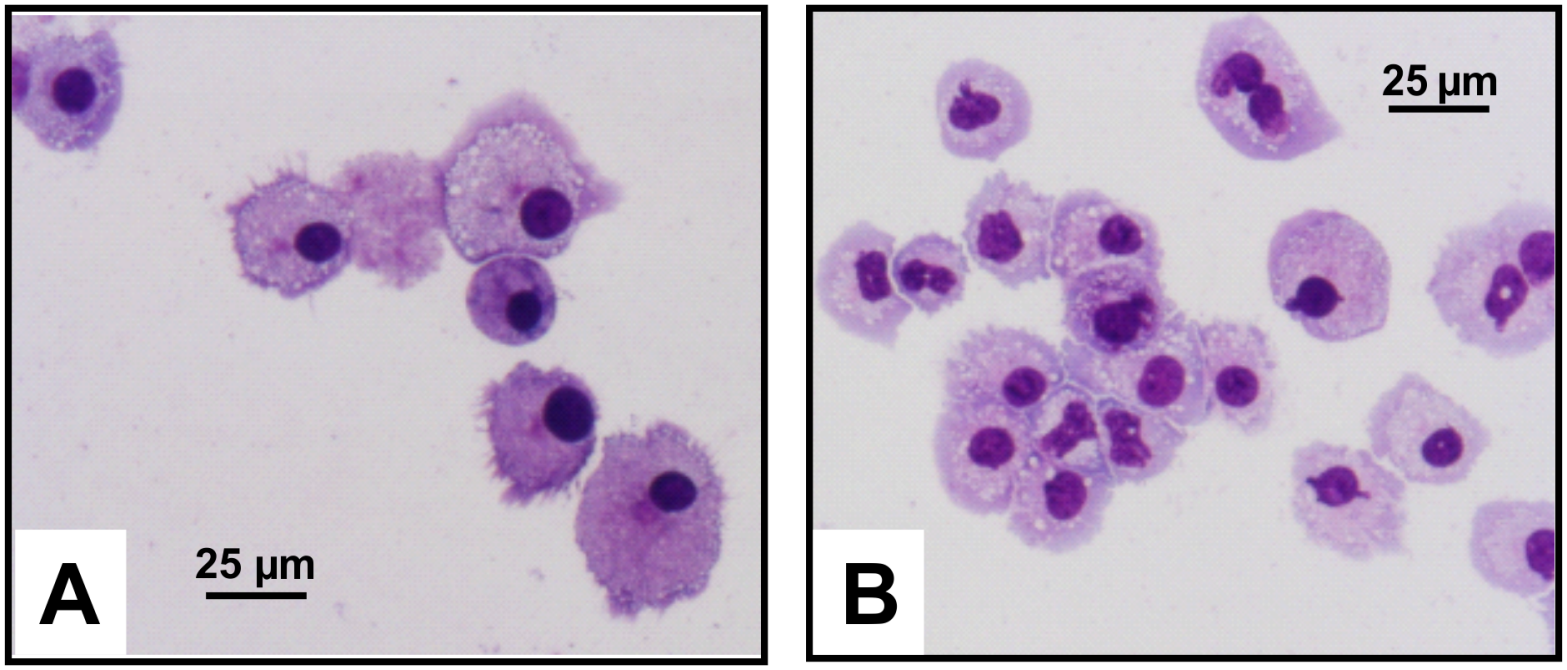
Peritoneal cell-derived and bone marrow-derived mast cells in primary culture. Cultured PMCs **(A)** and BMMCs **(B)** were concentrated on cytospin slides, stained with the fast stain dye, and analyzed by light microscopy (Original magnification 200x; bars, 25 μm).

MCs have a highly characteristic morphology that is mainly characterized by granules that contain a plethora of preformed constituents including lysosomal enzymes, biogenic amines, cytokines, proteoglycans, MC-specific proteases, non-MC-specific proteases, and membrane associated proteins [21]. The metachromatic granules in fixed MCs can be easily detected in the fast stain procedure (Fig 9).

In the staining protocol that we use in our laboratory, approximately 70,000 cells are first fixed on Shandon cytospins (#59910056/M, Thermo Scientific) by centrifugation following a standard procedure. After centrifugation, the cells were fixed in methanol, and subsequently stained with Haema fast staining solution for 5 min (#LT 003, LT-SYS Diagnostika, Berlin, Germany), and then stained for 10 min with solution LT 004 (LT-SYS Diagnostika). Thereafter, the stained cells were washed for 2 min in distilled water. Then the cells were air-dried and analyzed by light microscopy.

### Cell staining and FACS sorting

In our laboratory we use the Mast/stem cell growth factor receptor that is also known as proto-oncogene Kit (CD117) and the FcεRIα for identity verification. We use this double stain because the cell surface marker CD117 that mediates sensitivity towards SCF is also present on certain other types of hematopoietic progenitors, melanocytes, intestinal cells, some stem cells, and earliest lymphoid progenitors. On the other side, the high-affinity IgE receptor FcεRIα is not only found on MC but also detectable in eosinophils, basophils and epidermal Langerhans cells. The double stain, however, is only found in MCs and highly suitable to verify MC identity.

**Fig 10:**
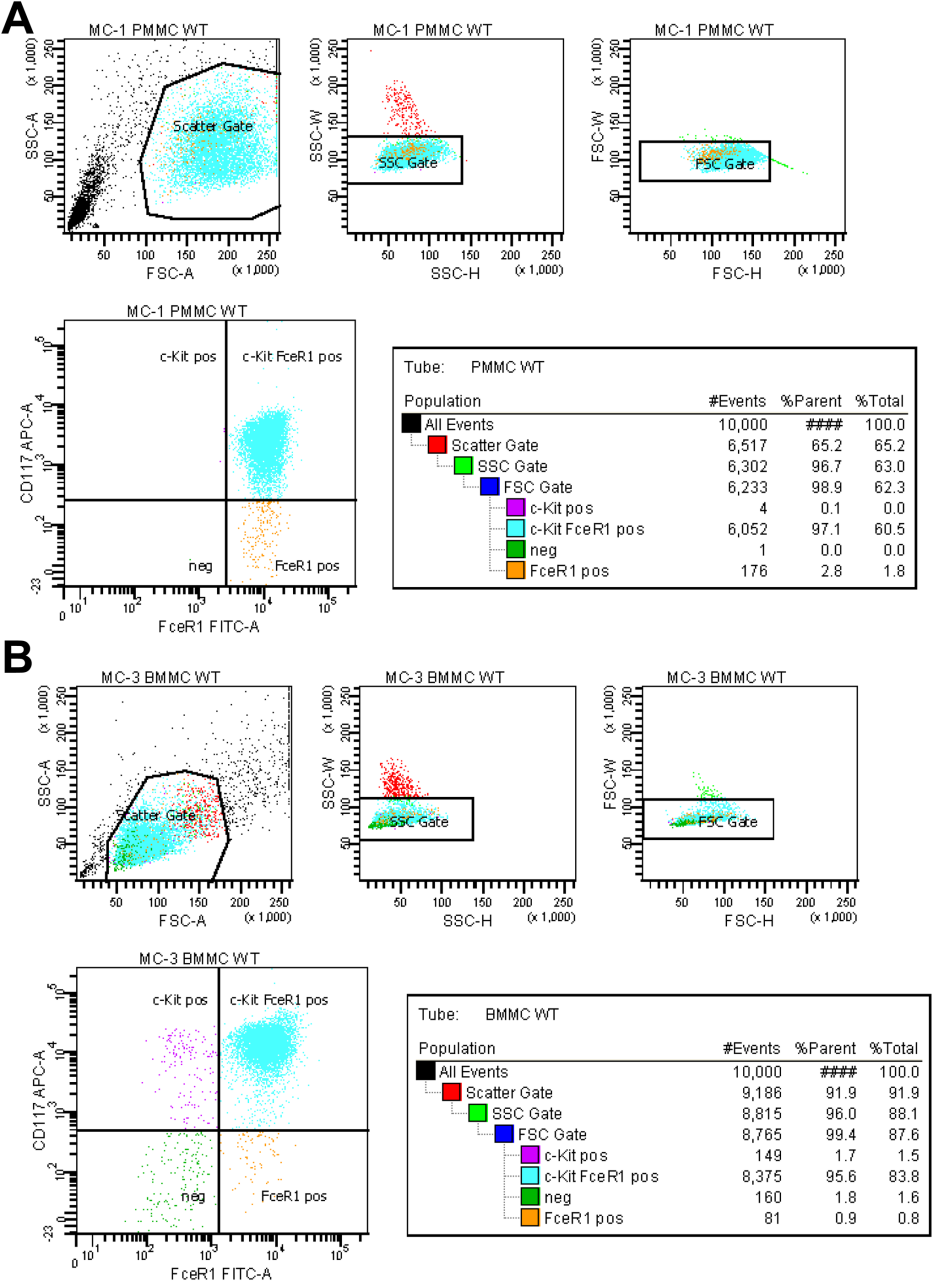
Analysis of surface markers. **(A)** PMCs and **(B)** BMMCs were stained for FcεRIα and Kit and analyzed by FACS. Side scatter (SSC) and forward scatter (FSC) in each sort are marked and the fraction of cells positive for Kit (*purple*), FcεRIα (*orange*) or both markers (*light blue*) indicated. Please note that in this sort, 97.1% of PMCs are positive for both markers, while only a small fraction of cells (2.8%) are positive for FcεRIα alone. In addition, 95.8% of all BMMCs were positive for both markers, while only 1.7% stained positive for Kit and 0.9% for FcεRIα alone.

In our flow cytometry, PMCs and BMMCs that were cultured for 3–4 weeks were incubated with anti-mouse CD117/KIT-APC (1:400) (BD Pharmingen, 553356) and anti-mouse FcεRIα-FITC (1:400) (eBioscience, 11-5898-82) that is specific for the high-affinity IgE receptor, α subunit. The labelled cells were washed twice with PBS, resuspended in PBS at a cell density of 1 x 10^6^/mL and analysed in a BD FACSAria II (Becton Dickinson, Heidelberg, Germany). This FACS machine is equipped with a laser with an excitation wavelength of 640 nm and 488 nm and bandpass filters (670/14 BP, 505 LP followed by 525/50 BP) that allows to detect the stained cells at the emission ranges of the fluorophores (Fig 10).

In principle, the expression of KIT and the FcεRI can also be proven by Western blot analysis. However, it should be mentioned that MCs and especially PMCs contain large quantities of diverse proteases that become released from sensitized or damaged MCs. In particular the large quantity of aggressive proteases in PMCs might hinder or even prevent the detection of individual proteins in Western blot analysis when samples are prepared by standard protein extraction methods [10].

## Conclusions

Over the past few decades, the functional importance of MCs as important effector cells of the immune system has been convincingly established. However, some of the biological attributes of MCs described were only established in immortalized cell lines resulting in findings that must be interpreted cautiously. The outlined protocol provides a rational for reliable isolation of primary mature peritoneal MCs and bone marrow-derived MC precursors from mouse. Both MC entities can be effectively expanded in culture and maintained for weeks with a phenotype that is characteristic for MCs (e.g. positivity for KIT and FcεRIα, protease expression in secretory lysosomes, and ability to degranulate). The simultaneous availability and comparative analysis of culture-maturated and tissue-derived MCs from one biological source will be helpful to dissect molecular pathways that are in general necessary for MC differentiation or to identify specific features of MCs derived from different origin. Furthermore, the parallel analysis of both MC entities will allow to set up studies in which the biological effects during various inflammatory settings can be comparatively analyzed. In addition, it will be essential to analyze if MCs isolated from different sources have the same biological attributes. In this context it should be mentioned that most recently it was shown that in the skin acupuncture points have a higher density of MCs compared with non-acupoints and that further the primo nodes isolated from the surfaces of internal organs (e.g., liver, small and large intestines, and bladder) as well as from the lymph vessels of rats contain relative high concentrations of MCs reaching values up to 15–20% of the residential cells with respect to other cells [22].

## Supporting Information

S1 Table Material list. (DOC)

## Abbreviations

BMMC(s): bone-marrow-derived mast cell(s)
MC(s): mast cell(s)
PMC(s): peritoneal cell-derived mast cell(s)
CTMC(s): connective tissue mast cell(s)
MMC(s): mucosal mast cell(s)
MCPT: mast cell protease
