# Supplementary material for: Isolation of Mature (Peritoneum-Derived) Mast Cells and Immature (Bone Marrow-Derived) Mast Cell Precursors from Mice
Source: PLoS One. 2016 Jun 23;11(6):e0158104. doi: 10.1371/journal.pone.0158104 (PMC4918956; doi:10.1371/journal.pone.0158104)
Supplement: S1 Table — (DOC) [file pone.0158104.s001.doc]

**Supplemental Table 1: Material List***

| **Material** | **Company** | **Catalogue number** | **Comments** |
| --- | --- | --- | --- |
| 10 mL syringe | Becton Dickinson Plastipak, Heidelberg, Germany | 309604 | disposable |
| Hypodermic needle (19 G), Microlance 3 | Becton Dickinson | 301500 | disposable |
| Cell culture hood | Heraeus, Kendro Laboratory Products, Langenselbold, Germany | Herasafe HSP 12 | Safety cabinet allows operation in safety levels 1 and 2. |
| Cell culture incubator | Heraeus | 51007918 | This incubator device is certified by the German Institute for standardisation (DIN 12880/1) |
| Centrifuge | Heraeus | Megafuge 1.0 R | tabletop centrifuge with refrigeration and equipped with |
| Swinging bucket rotor (4 x 400 mL) | Heraeus | 75002704 |  |
| Rectangular bucket | Heraeus | 75002252M |  |
| Adapter for swinging rotor | Heraeus | 75005324 |  |
| Cotton-swabs | Nobamed Paul Danz AG, Wetter/Ruhr, Germany | 974202 | disposable |
| Cotton Gauze swabs (20 x 20 cm) | Fuhrmann GmbH, Much, Germany | 32014 | disposable |
| Falcon tubes |  |  | disposable |
| Graefe forceps straight / serrated (0.8 mm-1.0 mm Tip) | Fine Science Tools Inc., Heidelberg, Germany | 11050-10 |  |
| Isoflurane | Forene Abbott, Wiesbaden, Germany | B506 | halogenated anesthetic gas, potential health hazard, do not store above 25^o^C |
| Plastic (PE) Transfer pipettes (7 mL), 3 mL graduated | Karter Scientific Labware Manufacturing, Lake Charles, LA, USA | 206H3 | disposable |
| Plastic serological pipettes (10 mL) | Greiner Bio-One, Frickenhausen, Germany | 607180 | disposable |
| Poly-Alcohol Haut…farblos Antisepticum | Antiseptica GmbH, Pulheim/Brauweiler, Germany | 72PAH200 | contains 70%(v) 2-propanol |
| Sterican needle 26 G x 1 | B. Braun Melsungen AG | 4657683 | disposable |
| Strabismus Scissor (straight, 11.5 cm length, 23 mm effective cutting) | Fine Science Tools Inc. | 14074-11 |  |

* given materials are those that we use in our laboratory. Alternative materials with similar properties provided by other supplieres are equally usable.
